# Short-term resveratrol treatment restored the quality of oocytes in aging mice

**DOI:** 10.18632/aging.204157

**Published:** 2022-07-08

**Authors:** Naoki Okamoto, Yorino Sato, Yuta Kawagoe, Takahiko Shimizu, Kazuhiro Kawamura

**Affiliations:** 1 Department of Obstetrics and Gynecology, International University of Health and Welfare School of Medicine, Chiba 286-8686, Japan; 2 Department of Biodesign, Institute of Biomaterials and Bioengineering, Tokyo Medical and Dental University, Chiyoda-ku, Tokyo 101-0062, Japan; 3 Department of Obstetrics and Gynecology, Juntendo University Faculty of Medicine, Bunkyo-ku, Tokyo 113-8421, Japan; 4 Aging Stress Response Project Team, National Center for Geriatrics and Gerontology, Obu, Aichi 474-8511, Japan

**Keywords:** resveratrol, age-associated infertility, oocyte quality, Sirtuin, mitochondria

## Abstract

The quality of oocytes declines by aging, resulting in their low competences for fertility. Here, resveratrol treatment showed increases in the rates of implantation and live offspring as well as decreases in the abortion rate as short as one week after treatment, although the number of ovulated oocytes and the rates of fertilization and blastocyst formation were not changed following resveratrol treatment. Resveratrol treatment did not cause abnormalities mouse estrous cycles and body weights. No abnormality was detected in both fetuses and placentas after 22 weeks of resveratrol treatment and the fetuses had normal fertility. Positive correlations were found between serum resveratrol levels and pregnancy and live offspring rates as well as ovarian expression levels of *Sirt1*, *Sirt3*, *Sirt4*, *Sirt5*, and *Sirt7*. The mitochondrial membrane potential and ATP content but not copy number of mitochondrial DNA in oocytes was increased in aging mice with resveratrol treatment. In conclusion, we demonstrated the restoration of oocyte quality in aging mice in addition to the prevention of their quality decline during aging by restoring mitochondrial functions by resveratrol treatment without adverse effects in the animals and their offspring.

## INTRODUCTION

The quality of oocytes declines by aging, resulting in their low competences for pregnancy and live birth [1–3]. Although the major causes of poor quality in oocytes following aging are chromosomal abnormality [4, 5], age-dependent increases in cellular [6] and DNA [7] damages induced by reactive oxygen spices, decreased mitochondrial copy number and ATP production [8] were also found. These abnormalities accumulated during aging and eventually lowered oocyte quality. Thus, it is important to develop methods to restore the oocyte quality in infertile women with advanced age to establish an anti-aging therapy.

Resveratrol is a type of plant polyphenol found in grape, red wine, peanuts etc. and available as a supplemental diet with the anti-oxidative and inflammatory actions [9]. Furthermore, resveratrol has demonstrated to activate Sirtuins implicated in anti-aging cellular processes and to promote mitochondrial functions [10]. In a recent study, anti-aging activity of resveratrol to prevent the decline of oocyte quality during aging was examined by feeding young mice with drinking water including resveratrol for 6 and 12 months [11]. This study revealed increases in litter size under natural mating in mice following 12 months resveratrol intake, but not after the 6 months treatment. At the molecular level, resveratrol intake decreased the expression of an aging marker *p21* in ovaries to levels comparable to those in young mouse counterparts [11]. Although this study demonstrated the prevention of aging-induced decline of fertility by a long-term resveratrol treatment from young age, such a long treatment starting from young age is not practical in clinical settings.

To establish an anti-aging therapy for women with advanced age, it is important to find a protocol with short-term treatment. Therefore, in order to develop the basis for future clinical application, we sought to determine the effect of a short-term resveratrol treatment on the restoration of reduced fertility in aging mice using models at different treatment period of resveratrol. We demonstrated increases in the rates of implantation and live pups as well as decreases in the abortion rate as short as one week after resveratrol treatment. We further found positive correlations between serum resveratrol levels and pregnancy and live pups rates as well as ovarian expression levels of *Sirt1*, *Sirt3*, *Sirt4*, *Sirt5*, and *Sirt7* as potential downstream anti-aging effectors. With increased mitochondria-related *Sirt3*, *Sirt4*, and *Sirt5* expressions, we demonstrated the restoration of mitochondrial function in oocytes following resveratrol treatment.

## RESULTS

### Resveratrol did not affect mouse estrous cycle and body weight

**Figure 1:**
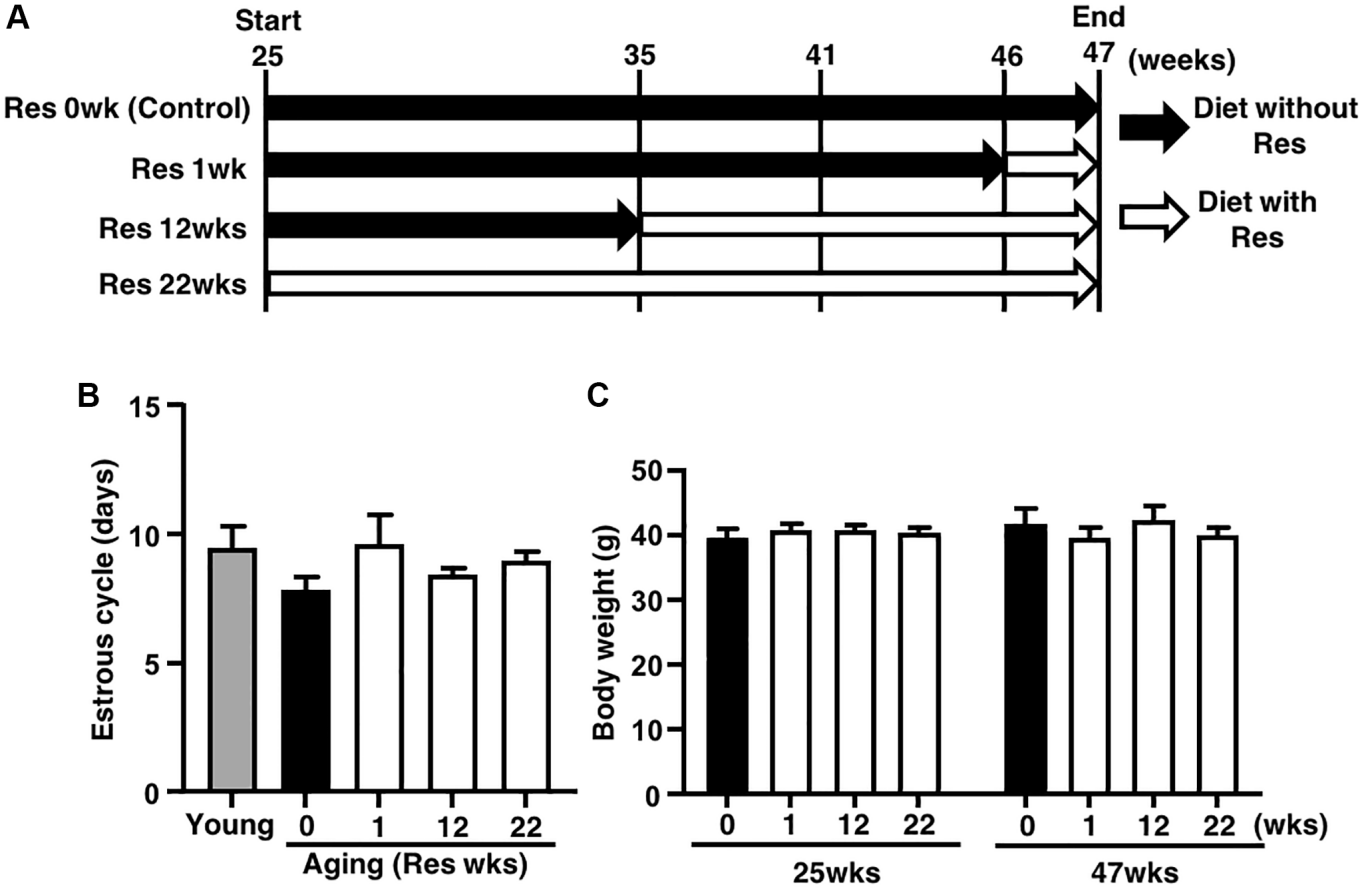
**Study design and effects of resveratrol (Res) treatment on estrous cycle and body weight during mouse aging.** (**A**) Forty ICR mice at 25 weeks (wks) of age were housed until 47 weeks of age and fed with or without Res. These mice were divided into four groups (10 mice in each group) depending on four different feeding durations: 0 (control), 1, 12 and 22 weeks. In addition, young mice served as controls in some experiments to confirm aging changes in reproduction. Mice were weighed and recorded at the start of resveratrol treatment (25 weeks of age) and at 47 weeks of age. After 47 weeks of age, ovulated oocytes were collected and then *in vitro* fertilization-embryo transfer was performed to determine the number of ovulated oocyte and the rates of fertilization, blastocyst formation, implantation, live pups and abortion. Some ovulated oocytes were used for the analyses of mitochondrial functions. (**B**) Estrous cycles during 22 weeks of treatment. Estrous cycles were evaluated using the smear of vaginal epithelial cells every 48 hours (*n* = 8–10 animals, *n* = 78 observations in each animal). (**C**) Body weights of each group at 25 and at 47 weeks of age at the start and end of resveratrol treatment, respectively (*n* = 8–10 animals). Bars represent means ± SE.

To assess the effect of resveratrol on ovarian follicle development, we determined changes of estrous cycle by vaginal smear of epithelial cells. As shown in Figure 1B, there was no difference in the average of estrous cycle pattern among four groups with different feeding period of resveratrol (0, 1, 12 and 22 weeks), suggesting no effect of resveratrol treatment on follicle growth. The animal body weights at 25 and 47 weeks of age were also measured to ensure that addition of resveratrol to the diet did not affect feeding behavior which could influence follicle development via body weight changes. We found that the body weights were not altered by resveratrol treatment and the weights were not increased after 25 weeks of age (Figure 1C).

### Resveratrol improved age-associated infertility

**Figure 2:**
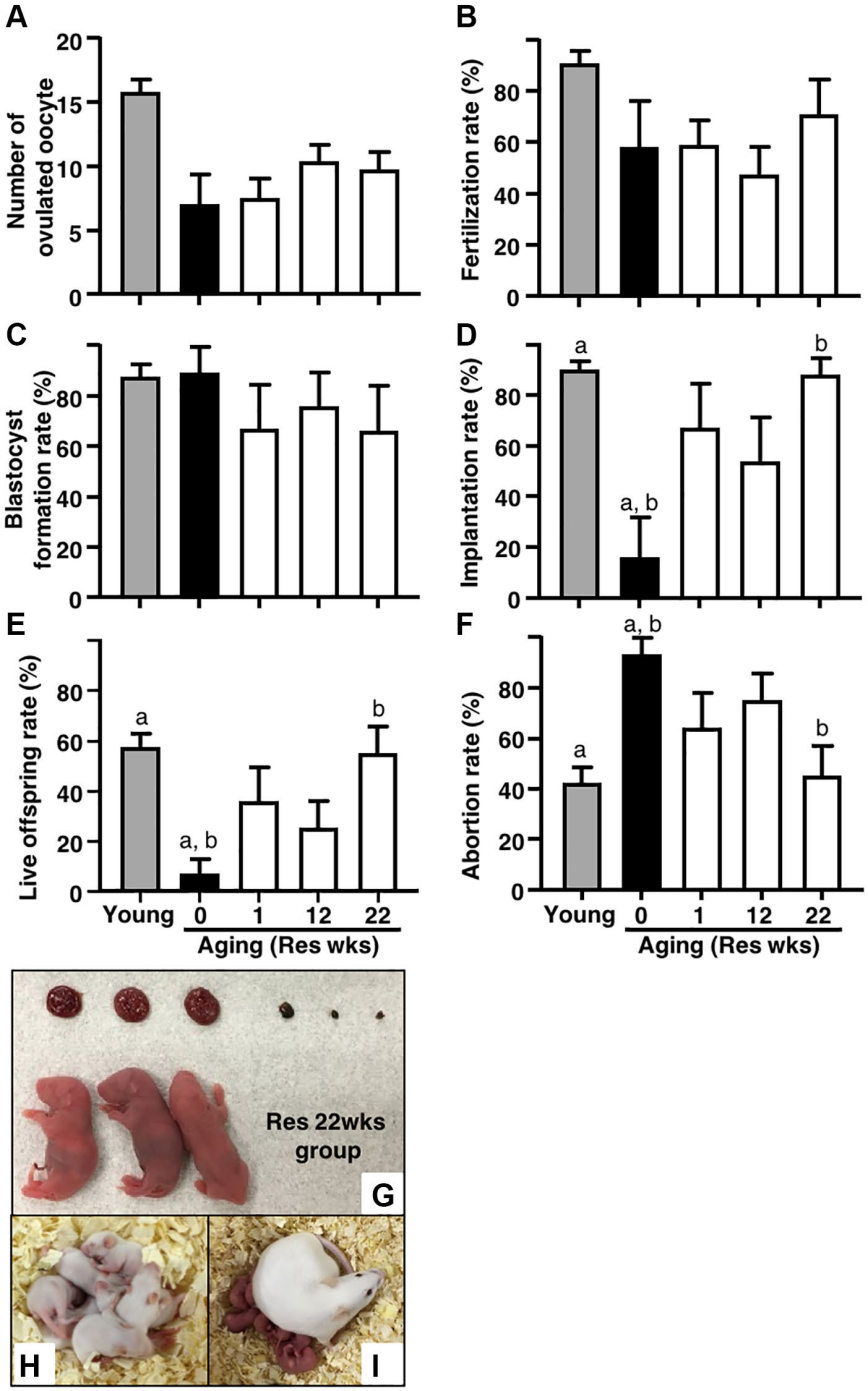
**Effects of resveratrol treatment on fertility in aging mice.** Ovulation was induced at proestrous stage after 47 weeks of resveratrol (Res) treatment by using hCG injection. At 15 hours after hCG administration, cumulus-oocyte complexes (COCs) were obtained from oviduct ampulla. COCs were inseminated with sperm collected from fertile male mice. At 16 hours after culture, 2-cell stage embryos were collected and allowed to develop to the blastocyst stage by additional 72 hours of culture. After embryo culture, blastocysts from each animal were transfer to independent recipient mouse. At 19 days after oocyte retrieval, Caesarian section was performed to count the number of implantation sites and live offspring. For young controls, ICR mice at 6 weeks of age were used. (**A**) Number of ovulated oocytes. The number of ovulated oocytes was determined by removal of cumulus cells surrounding oocytes after insemination under the stereomicroscope (*n* = 8 animals). (**B**) Fertilization rate (2-cell stage embryos/ovulated oocytes) (*n* = 6^*^–8 animals, 33–115.2-cell stage embryos per groups). ^*^, two mice in control group and one mouse in Res 22 weeks group did not ovulate. (**C**) Blastocyst formation rate (blastocysts/2-cell stage embryos) (*n* = 4^*^–8 animals, 28–105 blastocysts per groups). ^*^, oocytes retrieved from two mice in each control and Res 12 and 22 weeks group did not fertilize. (**D**) Implantation rate (implanted blastocysts/transferred blastocysts) (*n* = 4–7^*^ animals, 7–95 implanted blastocysts per groups). ^*^, 2-cell stage embryos derived from one mouse in each Res 1 and 22 weeks group were arrested to develop prior blastocyst stage. (**E**) live offspring rate (live offspring/transferred blastocysts) (*n* = 4–7 animals, 3–61 live offspring per groups). (**F**) abortion rate (1- live offspring/transferred blastocysts). (**G**) representative images of live offspring and placentas from Res 22 weeks group. After Caesarian section, the offspring were nursed by foster mothers to evaluate their healthiness and mated at 8 weeks of age to confirm their fertility. (**H**) the offspring at 10 days after Caesarian section, (**I**) the offspring with pups. Bars represent means ± SE. a, b *p* < 0.05 vs. controls.

For further insight into the potential of resveratrol treatment in the improvement of age-associated infertility, IVF-ET was conducted in the four groups of aging mice. Although aging mice without resveratrol treatment exhibited significantly reduced number of ovulated oocytes as compared with young counterparts, the number of ovulated oocytes in the resveratrol-treated groups was comparable to that in aging control mice (Figure 2A). The rates of fertilization and blastocyst formation were not declined by aging in our protocol and thus these was no room for improvement of these reproductive outcomes by the resveratrol treatment (Figure 2A–2C). After embryo transfer, the rates of implantation and live pups in aging mice without resveratrol treatment became <4-fold lower (Figure 2D and 2E) and the abortion rate became 2-fold higher than those in young animals (Figure 2D–2F). The resveratrol treatment dramatically improved these reproductive outcomes and those proportions in aging mice with a long-term treatment (22 weeks) reached similar levels as their young counterparts (Figure 2D–2F). Of note, the rates of implantation, live pups and abortion were also improved even in the group with a short-term treatment (one week) (Figure 2D–2F).

To confirm the safety of resveratrol treatment, gross morphology was evaluated in fetuses and placentas at Caesarean section. As shown in Figure 2G, no abnormal finding was detected in both live fetuses and corresponding placentas derived from embryos obtained from mice with 22 weeks of resveratrol treatment. The fetuses were further nursed by foster mothers and developed normally (Figure 2H: 10 days after Caesarian section). After mating, these mice delivered healthy pups (Figure 2I).

### Serum resveratrol levels correlated with implantation and live offspring rates and expression of ovarian Sirtuin family genes

**Figure 3:**
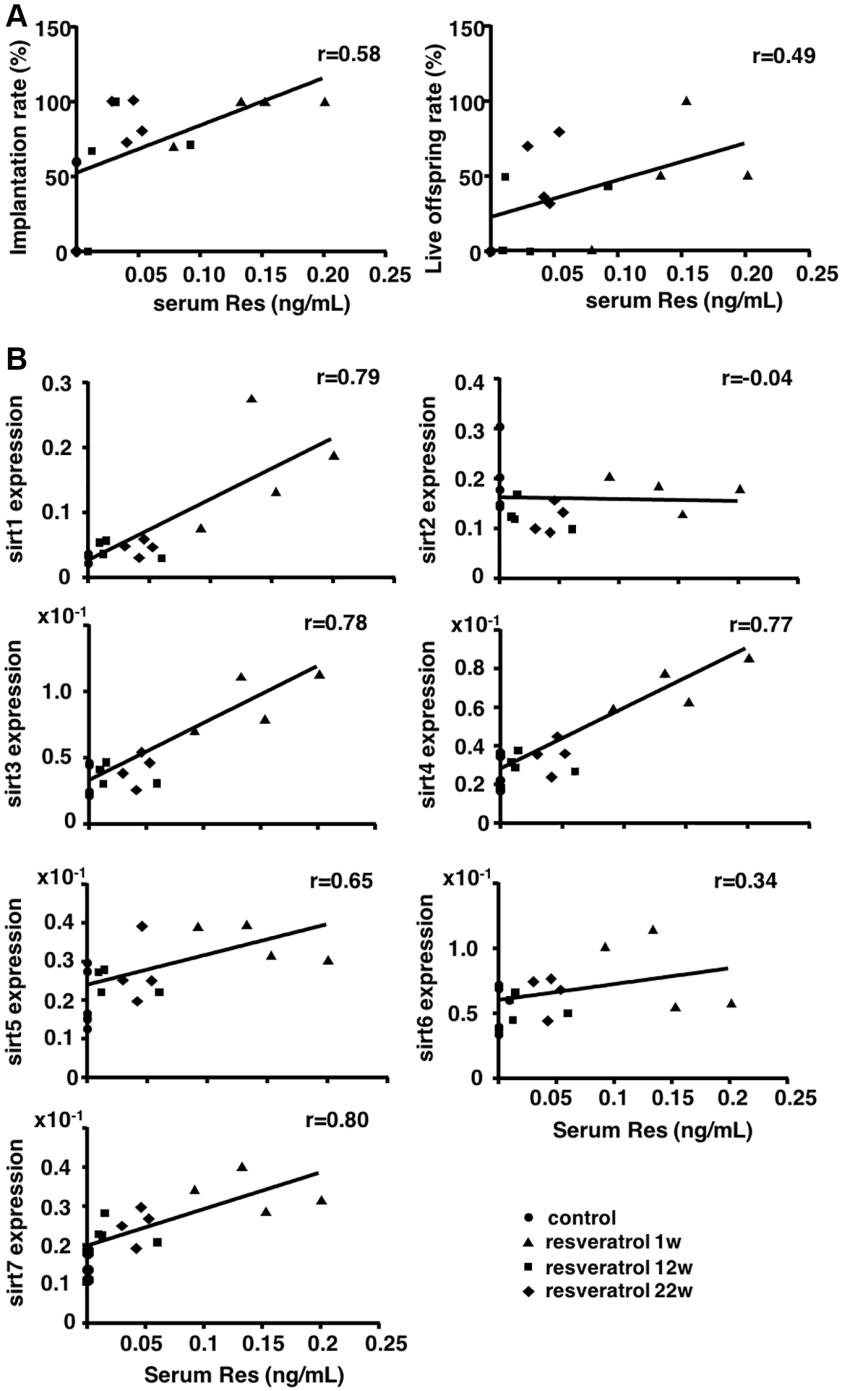
**Correlation between serum resveratrol levels and the rates of implantation and live offspring, and ovarian transcript levels of Sirtuin family.** Serum resveratrol (Res) levels from all groups were measured using HPLC-MS/MS. (**A**) Correlation of serum resveratrol levels with implantation and live offspring rates (*n* = 16 animals). (**B**) Correlation of serum resveratrol levels with ovarian mRNA expression levels of Sirtuin family genes (*n* = 16 animals). The correlation coefficient (r) above 0.4 indicated a significant correlation.

To evaluate the correlation between serum resveratrol levels and the rates of implantation and live offspring, the resveratrol levels in animals of all experimental groups were measured by high performance liquid chromatography (HPLC) -tandem mass spectrometry (MS/MS). After confirmation of the absence of resveratrol in serum without resveratrol treatment (aging controls), positive correlation was detected between serum resveratrol levels and implantation and live offspring rates (Figure 3A). Using same animals, correlation between serum resveratrol and ovarian Sirtuin family transcript levels was further analyzed. As shown in Figure 3B, the serum resveratrol levels were positively correlated with the expression levels of *Sirt1*, *Sirt3*, *Sirt4*, *Sirt5* and *Sirt7*, but not in *Sirt* 2 and *Sirt6*.

### Resveratrol improved mitochondrial functions in oocytes

**Figure 4:**
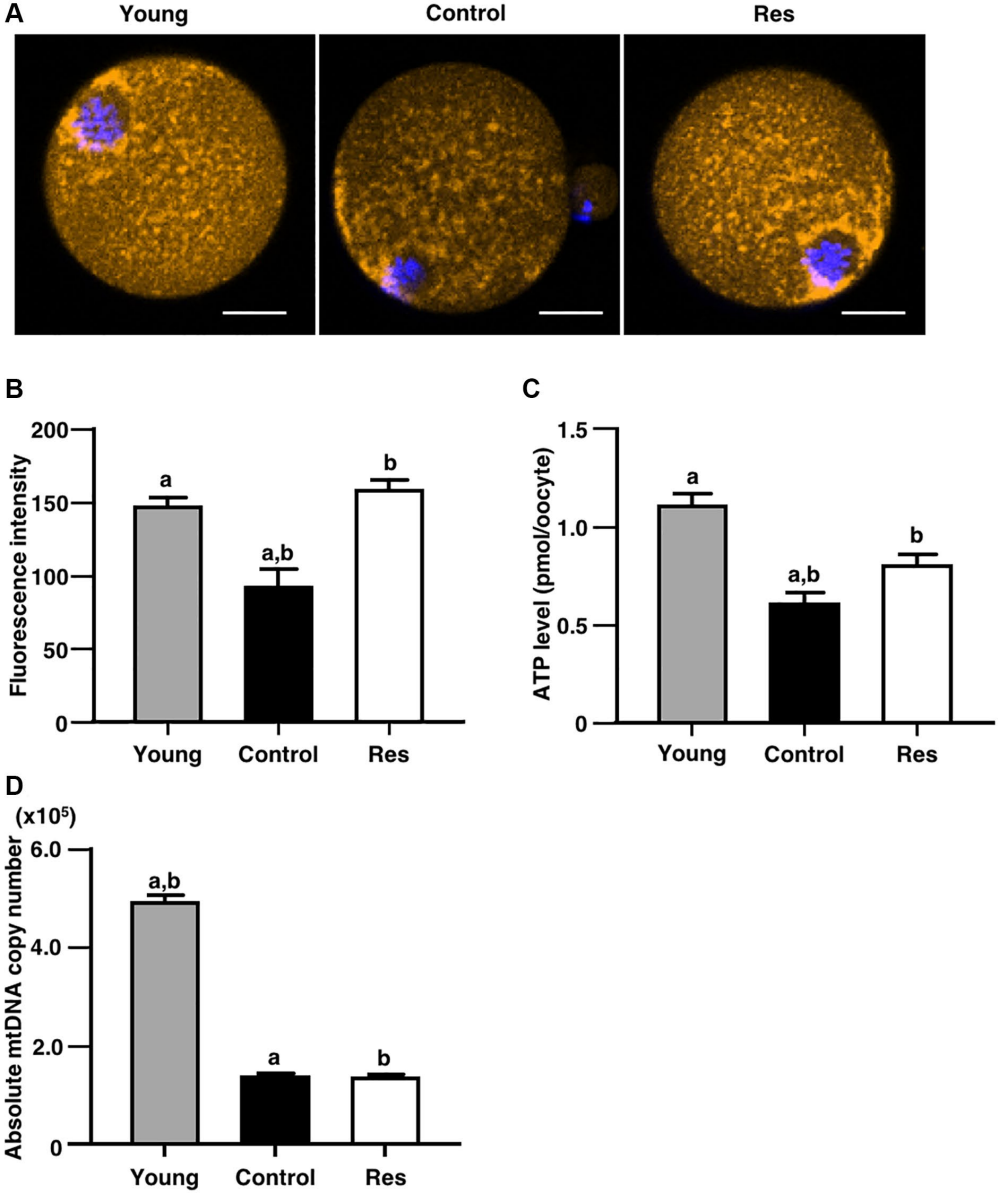
**Effects of resveratrol treatment on mitochondrial functions in oocytes derived from aging mice.** MII oocytes derived from aging mice without (control) or with one week of resveratrol (Res) treatment and young animals without resveratrol treatment (young) were used for different mitochondrial assays. (**A** and **B**) Mitochondrial membrane potential. (**A**) Representative fluorescence images showing mitochondrial membrane potential visualized by MitoTracker™ dye (orange). Oocyte nuclei were counterstained with Hoechst 33342 (blue). Scale bars, 20 μm. (**B**) The fluorescence intensities of mitochondrial membrane potential. The intensity of mitochondrial fluorescence in ooplasm of MII oocyte was measured by excluding that in the first polar body (control: *n* = 25, Res: *n* = 15 and young: *n* = 26 oocytes). (**C**) The ATP levels in MII oocytes. The ATP level per MII oocyte was measured using the ATP-Glo™ Bioluminometric Cell Viability Assay Kit (control: *n* = 24, Res: *n* = 16 and young: *n* = 17 oocytes). (**D**) The mitochondrial DNA (mtDNA) copy numbers of MII oocytes (10 oocytes from each group). The copy number was measured by absolute real-time RT-PCR. (control: *n* = 11, Res: *n* = 13 and young: *n* = 18 groups). Bars represent means ± SE. ^A, B^*p* < 0.05 vs. controls.

Due to presence of positive correlation with gene expression levels of mitochondria-related Sirtun families, the effects of one week of resveratrol treatment on mitochondrial functions of oocytes were determined. The mitochondrial membrane potential in oocyte was determined as an intensity of florescence signal by MitoTracker™ dye staining. As shown in Figure 4A and 4B, the intensity in oocytes derived from aging mice with the resveratrol treatment was significantly increased as compared with aging controls and recovered to the same levels of young counterparts. Furthermore, ATP content in oocytes was significantly increased by the resveratrol treatment (Figure 4C). The copy number of mitochondrial DNA in oocytes was declined by animal aging, but the resveratrol treatment did not improve those copy numbers (Figure 4D).

## DISCUSSION

Based on the *in vivo* study using animal models, we demonstrated the restoration of oocyte quality in aging mice following short-term resveratrol treatment. In addition, we confirmed that the long-term resveratrol treatment prevented the quality decline of oocytes during animal aging by restoring implantation and live offspring rates. Furthermore, we showed positive correlations between serum resveratrol levels and pregnancy and live pup rates as well as ovarian expression levels of *Sirt1*, *Sirt3*, *Sirt4*, *Sirt5*, and *Sirt7*. We also demonstrated the restoration of mitochondrial activity in oocytes derived from aging mice with the short-term resveratrol treatment.

Although one may consider clinical trials of resveratrol for the infertility treatment of aging women by extending earlier findings in aging mice [11]. These effects of resveratrol were evident if young mice at 6 weeks of age were treated with resveratrol for 12 months, but 6 months treatment did not exhibit such positive effects [11]. The design of this mouse study corresponded to resveratrol treatment from young girl (12.5–20 years-old) to menopause age in humans [12]. Such an early and long-term treatment from young age is not realistic for prevention of the aging-induced decline of fertility in women. Indeed, women likely start to consider future childbirth and fertility preservation at more advanced age and thus later and shorter treatment to improve fertility in aging women is important in infertility treatment.

Considering future clinical application, we designed new experiments to address restoration of oocyte quality in aging animals. Using the short-term resveratrol treatment, we succeeded to show the restoration of declined fertility in aging mice. Because the 1 and 12 weeks of treatment groups started resveratrol feeding from 46 and 35 weeks of age, respectively, the fertility of these animals was already declined at the initiation of resveratrol treatment and thus our data indicated the beneficial effects of resveratrol on recovering the declined quality of oocytes in aging mice. A recent study supports our data with demonstrating the improvement of survival rate of MII oocytes undergoing postovulatory aging by a short-term (15 days) intraperitoneal injection of resveratrol [13]. Because it takes around 3 weeks for follicle development from primordial to preovulatory stage in mice [14], the short-term resveratrol treatments likely targeted the developing secondary and antral follicles.

To assess the prevention of the aging-induced decline of fertility, animals at 25 weeks of age which corresponded to around 30 years-old women [12] was treated with resveratrol for 22 weeks as same duration in previous study [11], In contrast to the lack of significant improvement of live offspring rate in the group with 6 months resveratrol treatment in the previous study [11], we showed the improvement of implantation, live offspring and abortion rates in mice treated with resveratrol for 6 months. These conflict results might be caused by the difference of mouse age at the assessment of resveratrol treatment. Although we assessed the effects of resveratrol at about 48 weeks of age after 6 months treatment, the previous study designed to evaluate at 28 weeks of age in the group of 6 months treatment [11]. Because mice at 28 weeks of age unlikely exhibit the aging-induced decline of fertility, the previous study might fail to show the evident difference. From the above results, it is considered that resveratrol has both the effect of restoration of oocyte quality and prevention of quality deterioration on oocyte. Implantation rate and live birth rate are significantly higher depending on the treatment period of resveratrol. Therefore, the prevention effect is weak with short-term treatment, and long-term treatment may be necessary to obtain the effect.

In terms of the daily dose of resveratrol, we used the same resveratrol content of the diet according to a previous study showing the resveratrol-mediated suppression of age-dependent oxidative stress by inhibiting the generation of superoxide in other murine organs [15]. Although the study demonstrating the anti-aging activity of resveratrol to prevent the decline of oocyte quality during aging [11] allowed the mice to access freely to water including 30 mg/l resveratrol, the amount of resveratrol consumed by individual mice was unknown and the serum levels of resveratrol in mice were not determined. Here, we found a positive correlation between serum resveratrol levels and the pregnancy and live offspring rates, and *Sirt1*, *Sirt3*, *Sirt4*, *Sirt5*, and *Sirt7* transcript levels in the ovaries. These data suggested the improvement of fertility competence of aging mice through induction of ovarian Sirtuin expression by resveratrol treatment and could contribute the determination of optimal dose for future clinical application.

Among different Sirtuin genes, we showed the increase in *Sirt1*, *Sirt3*, *Sirt4*, *Sirt5*, and *Sirt7* expressions. Because *Sirt3*, *Sirt4*, and *Sirt5* are expressed in mitochondria maintaining energy homeostasis and regulating gene expression in response to cellular stress such as oxidative stress [16–18], we focused on the mitochondrial functions in oocytes for further insight into molecular mechanism underlying resveratrol actions. Herein, we found the improvement of mitochondrial activity and ATP production in oocytes obtained from aging mice with short-term resveratrol treatment as one of the factors for restoration of fertility. Sirt1 shows anti-aging activity by maintaining intracellular metabolism and DNA repair [19, 20], whereas Sirt7 reduces apoptosis and improves resistance to oxidative and genotoxic stress [21]. Thus, increases in Sirt1 and 7 levels might also associate with resveratrol actions on restoring oocyte quality in aging mice.

Similar to the previous study [11], we found that the number of ovulated oocytes was markedly reduced by aging but was not changed by resveratrol treatment in aging mice. We found the beneficial effects of resveratrol treatment on the restoration of implantation, live offspring, and abortion rates. Due to the absence of age-associated decline in the rates of fertilization and blastocyst formation in aging ICR strain mice, the positive effects of resveratrol on these rates were not evident in this study. Although the earlier study did not evaluate the effects of resveratrol on fertilization, embryo development and implantation, the effect of resveratrol treatment on fertility was examined using a mating test by comparing the litter size per plugs after mating and per pregnancies determined by the presence of apparent large abdomen at embryonic day between 16 and 17 [11]. In both 6 and 12 months of treatment groups, the litter sizes per pregnancy were larger than those per plug, suggesting some oocytes did not achieve pregnancies even if the mating were successful. This pregnancy failure could be caused by unsuccessful fertilization, embryo growth and implantation as well as miscarriage at early stage. Recent reports suggest that resveratrol may suppress decidualization of human endometrial stromal cell lines and potentially induce implantation failure [22]. However, in this study, since the foster mother is used for the analysis of implantation rate and live birth rate, there is no effect of resveratrol on the endometrium. On the other hand, maintaining high ATP levels in human embryos has also been reported to correlate with good development and implantation rates [23]. Therefore, it is considered that the improvement of implantation rate and live birth rate is the result of the positive effect of resveratrol treatment on the quality of oocyte.

In terms of the safety of resveratrol treatment, no adverse event was reported in other studies working on humans [24, 25], mice [11, 26, 27], and rats [28] even if the similar amount of ingestion [15, 29]. In this study, resveratrol treatment did not alter animal body weights and no prominent abnormality was detected during breeding. Furthermore, we confirmed the normality of offspring up to the third generation after resveratrol treatment using the IVF-ET analyses. These data indicated the safety of resveratrol treatment for anti-aging of oocytes.

Since resveratrol treatment restored the quality of oocyte in aging mice, it is expected to contribute to the recovery of fertility in infertile patients with advanced age. Moreover, our and previous studies revealed a potential of resveratrol treatment for the prevention of quality decline of human oocytes during aging in young women who wish future pregnancy. Based on these successful outcomes in animal studies, future randomized controlled trials comparing placebo diet could conclusively demonstrate the efficacy of resveratrol treatment in patients.

## MATERIALS AND METHODS

### Animals

Male and female ICR mice were purchased from CLEA Japan, Inc. (Tokyo, Japan). The mice were housed at a temperature of 22°C and humidity of 55% with a 12-hour light/12-hour dark cycle, and were allowed free access to food and water. In addition, estrous cycles were checked by smear of vaginal epithelial cell on every two days. Animals were handled and housed in accordance with the procedures specified by the Department of Animal Experiments at International University of Health and Welfare School of Medicine (Narita, Japan). All animal experiments were approved by the Animal Care and Use Committee at International University of Health and Welfare School of Medicine (19002NA).

### Protocol for resveratrol treatment

Forty female ICR mice at 25 weeks of age were randomly divided into four groups (each *n* = 10), and housed in five mice per cage. These mice were fed with diet (6 g per day) containing 0.04% (w/w) resveratrol (Tokyo Chemical Industry Co., Ltd., Tokyo, Japan) (resveratrol diet: RD), as described previously [15, 29], or not (control diet: CD). These four groups were classified based on the duration of resveratrol feeding (0, 1, 12 and 22 weeks): 1) controls fed with CD during whole breeding period, 2) fed with CD until 46 weeks of age and then fed with RD for one week, 3) fed with CD until 35 weeks of age and then fed with RD from 12 weeks and 4) 22 weeks of resveratrol treatment group fed with RD (Figure 1A). Because most ICR mice stopped ovulation at about 50 weeks of age in the preliminary survey (data not shown), these mice were too old for this assay. Thus, we used mice at 47 weeks of age with ovulations to analyze the anti-aging effects of resveratrol on reproduction. Some animals died during long breeding time and did not reach 47 weeks of age (control; *n* = 2, 1 week of resveratrol treatment; *n* = 2, 12 weeks of resveratrol treatment; *n* = 2). Resveratrol treatment was started from 25 weeks of age and continued for 22 weeks with same duration of treatment as described in the previous study [11]. Some animals had shorter treatment period for 12 weeks (about the half duration of 22 weeks) and one week to assess the prevention of quality decline in oocytes during aging.

### Mouse physical examinations

To evaluate estrous cycles, all mice were checked by smear of vaginal epithelial cell every two days. Body weights were measured at the start (25 weeks of age) and the end (47 weeks of age) of experiments. At the end, the animal number in groups of control, 1 and 12 weeks of resveratrol treatment decreased to eight.

### IVF-ET

The estrous cycle was checked every day in all mice when they reached 47 weeks of age. Then, the mice at proestrous stage received an intraperitoneal injection of gonadotropin (10 IU; ASKA Pharmaceutical, Tokyo, Japan). Because two mice remained at diestrous stage (constant diestrous) in the group of 22-weeks resveratrol treatment, these animals could not be used for ovulation induction. At 15 hours later, mice were euthanized, and cumulus oocyte complexes (COCs) were collected from the oviductal ampulla. COCs were then placed in 100 μl of TYH medium (LSI Medience Corporation, Tokyo, Japan) with sperm (3 × 10^5^ /ml). The sperms were collected from male ICR mice at 10 to 12 weeks of age and incubated in TYH medium for 10 minutes at 37°C under 5% CO^2^/95% air to complete their capacitation.

After 5 to 6 hours culture for fertilization, the inseminated oocytes were collected and transferred in a 30 μl drop of KSOM medium (Merck Millipore Corporation, Tokyo, Japan) under mineral oil (Irvine Scientific Sales Company Inc., Saitama, Japan), and incubated at 37°C for 24 hours. Then, fertilized embryos at two-cell stage were selected and cultured for additional 72 hours to form blastocyst. The fertilization rate was determined based on 2-cell stage embryos/ovulated oocytes, whereas blastocyst formation rate was measured as blastocysts/2-cell stage embryos.

After culture, the blastocysts derived from each animal were transferred to the uterus of pseudo-pregnant recipient ICR mice at 6 to 10 weeks of age. Caesarean section was performed at 16 days after embryo transfer, and the number of implantation sites and live fetuses were counted. The implantation rate was determined as implanted blastocysts/transferred blastocysts, whereas live offspring and abortion rates were calculated as live fetuses/transferred blastocysts and 1- live offspring/transferred blastocysts. To ensure the safety of resveratrol treatment, we checked gross morphology of placentas and fetuses during Caesarean section. The offspring were nursed by foster mothers and mated at 8 weeks of age to check for their fertility.

### Real-time RT-PCR for measurement of Sirtuin gene expression in ovary

Ovaries were obtained from mice after oocyte retrieval and five to six ovaries were randomly selected from each group for real-time RT-PCR analysis. Total RNA was extracted using a RNeasy Mini kit (QIAGEN Sciences, Valencia, CA, USA), and then cDNA was synthesized using a PrimeScript™ RT Master Mix (Takara, Tokyo, Japan) according to the manufacturer’s protocol. Quantitative real-time RT-PCR was performed using a Power SYBR^®^ Green Master Mix (Thermo Fisher Scientific, Waltham, US) by a SmartCycler (Takara) as described previously [30, 31]. The protocol for real-time PCR was as follows: 15 minutes at 95°C and then 45 cycles of 15 seconds at 95°C and 60 seconds at 60°C. The primers used are shown in Table 1. To determine the absolute copy number of target transcripts, cloned plasmid cDNAs for individual gene were used to generate a calibration curve. Purified plasmid cDNA templates were measured, and copy numbers were calculated based on absorbance at 260 nm. A calibration curve was created by plotting the threshold cycle against the known copy number for each plasmid template diluted in log steps from 10^5^ to 10^1^ copies. Each run included standards of diluted plasmids to generate a calibration curve, a negative control without a template, and samples with unknown mRNA concentrations. Data were normalized based on histone H2a transcript levels. Triplicate measurements were performed in one sample and the mean values were used for data analyses.

**Table 1:** List of primers for real-time RT-PCR.

**Gene**	**Forward primer**	**Reverse primer**
Sirtuin1	CCTTGGAGACTGCGATGTTA	GTGTTGGTGGCAACTCTGAT
Sirtuin2	GCAGTGTCAGAGCGTGGTAA	CTAGTGGTGCCTTGCTGATG
Sirtuin3	CTGACTTCGCTTTGGCAGAT	GTCCACCAGCCTTTCCACAC
Sirtuin4	GCTTGCCTGAAGCTGGATT	GATCTTGAGCAGCGGAACTC
Sirtuin5	AGCCAGAGACTCAAGACGCCA	AGGGCGAGCTCTCTGTCCACC
Sirtuin6	TCGGGCCTGTAGAGGGGAGC	CGGCGCTTAGTGGCAAGGGG
Sirtuin7	GGCACTTGGTTGTCTACACG	GTGATGCTCATGTGGGTGAG
Histon-H2a	ACGAGGAGCTCAACAAGCTG	TATGGTGGCTCTCCGTCTTC

### Analysis of membrane potential of oocyte mitochondria

Additional animals at 25 weeks of age were treated with or without resveratrol for 1 week for mitochondrial analysis (each *n* = 20). Oocytes were collected from ovulated COCs by removing cumulus cells under mechanical pipetting after 1–2 min of 300 μg/ml hyaluronidase (Merck, Darmstadt, Germany) treatment. For young control, oocytes were obtained from ICR mice at 6 weeks of age using same procedure (*n* = 5).

The oocytes were incubated with a MitoTracker™ Orange (Thermo Fisher Scientific) followed by nuclear staining using a Hoechst 33342 dye (Thermo Fisher Scientific) according to the manufacturer’s protocol (control: *n* = 25, resveratrol treatment: *n* = 15, and young control: *n* = 26). After incubation, the membrane potential of mitochondria was visualized by a confocal laser microscope (ZEISS, Oberkochen, Germany), and the fluorescence intensity was measured using the Zen imaging software (ZEISS).

### Analysis of oocyte ATP content

The ATP content in MII oocyte was determined by a ATP-Glo™ Bioluminometric Cell Viability Assay Kit (Biotium, San Francisco, USA) according to the manufacturer’s protocol (control: *n* = 24, resveratrol treatment: *n* = 16, and young control: *n* = 17). Individual oocyte was lysed, and its luminescence was measured immediately using a luminometer (Roche, Basel, Switzerland).

### Analysis of mitochondrial DNA copy number

Mitochondrial DNA copy number was also determined by real-time PCR according to the previously published method with modification [32] (control: *n* = 11, resveratrol treatment: *n* = 13, and young control: *n* = 18). Briefly, a MII oocyte was placed in Tyrode solution (Merck, Darmstadt, Germany) to remove the zona pellucida and first polar body. Each oocyte was loaded in a PCR tube with 6 μl lysis buffer (20 mM Tris, 0.4 mg/ml proteinase K, 0.9% Nonidet-40 and 0.9% Tween 20) and incubated for 2 hours at 55°C. Proteinase K was then inactivated by heating the samples for 10 minutes at 95°C, and subjected to the PCR analysis directly. Quantitative real-time PCR was performed using a Power SYBR^®^ Green Master Mix with previously established probe (B6) and primers (B6-forward and reverse) designed for specific amplification of mouse mtDNA [32]. To generate the standard curve for quantification, PCR products amplified with B6 forward and reverse primers were ligated into T-vector. Twenty five-, 50- and 100-fold serial dilutions of purified plasmid standard DNA were used to generate the standard curve. Triplicate measurements were performed in one sample and the mean values were used for data analyses.

### Measurement of serum resveratrol levels

Blood was obtained from the heart using 1 ml syringe with 25 G needle immediately after euthanasia for oocyte retrieval. Then, serum sample was collected as a supernatant after centrifugation of blood at 900 g for 10 minutes at room temperature. Because more than 500 ml of serum was required to measure resveratrol levels using HPLC (Nexera X2 system controlled by CBM-20A, Shimadzu corporation, Kyoto, Japan)- MS/MS (triple quadrupole AB-Sciex model API 5000 mass spectrometer, AB-Sciex, Ontario, Canada), some animals without sufficient serum samples were excluded from the study.

To prepare the sample for HPLC-MS/MS, 10 μl of the internal standard solution (25 ng/ml Trazamide, Fujifilm Wako pure chemical corporation, Osaka, Japan) and 10 μl borate buffer (pH 9.18) were added to 50 μl of each serum sample and the mixture was stirred for 10 seconds. Then, 800 μl of ethyl acetate was added to the mixture. After 3 minutes stirring, the mixture was centrifuged at 4°C for 2 minutes by 5,000 × g. The organic layer was separated into a glass tube and evaporated to dryness under a nitrogen stream at 40°C. Fifty μl of methanol was added to the residue and the mixture was stirred for 30 seconds followed by sonication for 1 minute to dissolve. One hundred fifty μl of water was added to the mixture and stirred for 30 seconds. After a centrifuge at 4°C, for 3 minutes by 2,000 × g, the supernatant was transferred into the HPLC-MS/MS system.

The identification and quantification of resveratrol and its metabolites in serum was performed by HPLC-MS/MS according to the manufacture’s protocol. Serum samples were analyzed by HPLC separation using a CAPCELL PAK C18 MG II column (Shiseido, Tokyo, Japan) on HPLC. A 10 mM ammonium acetate solution was used for mobile phase A, and methanol was used for mobile phase B. These samples were transferred into the column maintained at 40°C. Mobile phases A and B were eluted at a flow rate of 0.3 ml/minute with a linear gradient in which the volume ratio was changed from 80:20 to 0:100. The gradient elution was performed as follows: 20% B (0–0.5 minuets), 80% B (0.5–6.5 minutes), 100% B (6.51–7.5 minutes), and then 20% B (7.5–10.5 minutes). The system equipped with an electrospray ionization source and operated in the negative ion mode with multiple reaction monitoring mode. Samples were analyzed in negative ion mode with the tune method set as follows: collision gas (nitrogen) flow rate of 6 arb, curtain gas (nitrogen) flow rate of 10 arb, nebulizer gas (air) flow rate of 60 arb, desolvation gas (air) flow rate of 60 arb, Ion spray voltage of 4.5 kV, entrance potential of 10 V, and collision cell exit potential of 10 V. The monitor ion ranges were m/z 227–143 (Res) and m/z 310–170 (trazamide).

### Statistical analysis

The results were expressed as the mean ± standard error. One-way ANOVA test was used as an intergroup comparison and Dunnett’s test used for multiple comparisons. The level of significance was set at *p* < 0.05. The serum resveratrol level was expressed as correlation with either Sirtuin mRNA expression level, implantation rate or live offspring rate. The correlation was analyzed using Pearson’s correlation coefficient. The correlation coefficient (r) above 0.4 indicated the significant correlation.
